# Association of the Healthy Eating Index with Metabolic Abnormalities among Middle-Aged Adults Living in Single-Person Households in Korea

**DOI:** 10.3390/nu13113937

**Published:** 2021-11-04

**Authors:** Yun-Jung Bae, Kwang-Won Yu, Kyung-Haeng Lee, Keum-Il Jang

**Affiliations:** 1 Major in Food and Nutrition, Division of Food Science and Biotechnology, Korea National University of Transportation, Jeungpyeong 27909, Korea; kwyu@ut.ac.kr (K.-W.Y.); leekh@ut.ac.kr (K.-H.L.); 2 Department of Food Science and Biotechnology, Chungbuk National University, Cheongju 28644, Korea; jangki@chungbuk.ac.kr

**Keywords:** Healthy Eating Index, metabolic abnormalities, single-person, Korean

## Abstract

This study aimed to analyze the association between the dietary lifestyles and health outcomes among middle-aged (40–64 years old) and elderly (65 years old and older) individuals living alone using the Korean Healthy Eating Index (KHEI). The study was conducted with 1442 participants (475 men and 967 women) aged 40 years and older living in single-person households using the Korea National Health and Nutrition Examination Survey from 2016 to 2018. The KHEI scores were calculated based on the 24-h recall data of dietary intake. Among women living alone, the total KHEI score of the participants aged 40–64 years was 65.92, which was significantly lower than the 70.66 of those aged 65 years and older (*p* = 0.0152). In addition, the total score in the adequacy domain was significantly lower among the 40~64-year-old group than those aged 65 years and older (*p* = 0.0011). Among the elderly in single-person households, the odds of diabetes in the T1 group were 2.08 times higher than those in the T3 group according to the KHEI (95% confidence interval: 1.36–3.17). The results of this study are expected to be used as baseline data to establish nutrition, home meal replacement utilization, and health policies for the elderly living alone.

## 1. Introduction

Health varies by sex, education, household income level, marital status, as well as regional differences. Moreover, lifestyle habits, such as drinking, smoking, dietary supplement use, and physical activity, are also closely related to health [1,2]. Although the global average life expectancy has increased, its gap with healthy life expectancy has not decreased [3]. Thus, there is a growing interest in identifying factors that may increase healthy life expectancy.

Among many health-related factors, household ones are increasingly identified as important [4,5]. Individuals living together, such as families with children, share the household structure, the expertise in planning and preparing meals, and income, which highlights the close relationship between household factors and various health problems. For example, household food insecurity has been associated with poor diet quality and obesity [6,7], and single-person households have also exhibited a wide range of health and nutrition issues [4,8].

The dietary life of single-person households is characterized by a preference towards processed and convenient foods, as well as eating out to minimize time, physical, and mental efforts rather than purchasing ingredients and preparing meals [9,10]. Moreover, the income uncertainty of single-person households leads to a reduction in meal expenses and health management expenditure [11]. Studies have shown that these households are more likely to lack nutritional information and have difficulty maintaining a balanced diet compared to multi-person households [12]. Additionally, meals in single-person households often lack variety and specific core foods, such as fruits, vegetables, and fish, and are associated with unhealthy dietary patterns [13]. In Japan, men aged 60 years and older who were living alone had significantly lower fruit and vegetable intake and a poor overall diet [14]. Furthermore, single-person households with poor habits, such as erratic lifestyle, irregular meals, smoking, and drinking, may increase the risk of adverse health outcomes [15,16,17]. In a prospective cohort study conducted in Germany, living alone was an independent risk factor for mortality in men [17], and, in a study of Japanese men aged 65 and older, the risk ratio of obesity was significantly higher in single-person compared to multi-person households [18]. In addition, previous studies reported that the incidence of type 2 diabetes significantly increased among middle-aged adults living alone [19,20]. As such, single-person households are more likely to have poor dietary lifestyles, and there are growing concerns about their potential health implications.

In Korea, adults living alone are more likely to skip breakfast and less likely to have healthy desserts and traditional meals compared to those living with others [21]. In addition, among adults aged 65 years and older, single-person households had a significantly lower intake of almost all nutrients and a higher intake of insufficient nutrients than multi-person households when compared to the Korean dietary reference [8].

Recently, there have been changes in living arrangements worldwide due to changes in the social structure [22]. This has led to the rapid rise of single-person households in Korea from 27.2% in 2015 to 30.2% in 2019 (Statistics Korea 2020). The ratio of single-person households in Korea also varies according to sex and age. It was relatively higher among women aged 60 years and older and men in their third to the fifth decade of life [23].

Despite these recent changes in single-person household patterns, previous diet and health studies have mostly focused on the elderly [5,8,24] and adults aged 19 years and older [21,25]. A few studies have investigated the relationship between dietary factors and metabolic abnormalities in single-person households among individuals aged 40 and older. This is necessary because health management at this age is crucial for a healthy life in older age, and it is when metabolic abnormalities, such as hypertension, diabetes, and obesity, become more apparent.

This study aimed to identify the association between Korean Healthy Eating Index (KHEI) scores, which considers the characteristics of Korean meals and national dietary guidelines, and metabolic abnormalities in middle-aged adults living in single-person households.

## 2. Materials and Methods

### 2.1. Data Source and Study Population

The data used in this study were extracted from the Korea National Health and Nutrition Examination Survey (KNHANES) from 2016 to 2018, which was carried out by the Korea Centers for Disease Control and Prevention (KCDC). KNHANES is a complex, stratified, multistage probability, and cluster survey, conducted year-round in a rolling method to sample participants representing the Korean population. KNHANES consists of health interviews, examinations, and nutrition surveys in a cross-sectional design. Details regarding the KNHANES are described in the study by Kweon et al. [26]. The survey protocol was approved by the Institutional Review Board of the KCDC (approval no. 2018-01-03-P-A), and all participants provided informed consent.

Participant selection was based on several factors. Among the 11,727 who were aged 40 years and older who participated in all three surveys, those with missing data in the KNHANES (*n* = 17) and abnormal dietary intake (men with <800 kcal/day or >4000 kcal/day, women with <500 kcal/day or >3500 kcal/day) (*n* = 447) were excluded [27]. Participants whose data did not include the variables of obesity prevalence, hypertension, and diabetes (*n* = 495), dependent variables of this study, and those with missing socioeconomic characteristics (*n* = 488) were also excluded. Among the remaining participants, only those living in single-person households were included.

### 2.2. Data Collection

#### 2.2.1. Socio-Demographic Factors and Metabolic Abnormalities

Socio-demographic factors in the analysis included age, sex, education level, household income, marital status (married or single), drinking status (drinking at least once a month in the past year), smoking status (having smoked at least 100 cigarettes in a lifetime and currently smoking every day or some days), and exercise (walking for at least 30 min 5 days per week in the recent past). Respondents who answered “none” to the “How many people live with you in this household?” (variable: cfam) were classified as single-person households.

Health examination data were used for variables related to obesity, diabetes, and hypertension. Obesity was classified by body mass index (BMI) into underweight (BMI < 18.5 kg/m^2^), normal weight (18.5 ≤ BMI < 23 kg/m^2^), overweight (23 ≤ BMI < 25 kg/m^2^), and obese (BMI ≥ 25 kg/m^2^) [28]. Diabetes was determined by a fasting blood sugar of ≥126 mg/dL, self-reported diagnosis by a physician, and/or use of hypoglycemic medications or insulin. Hypertension was defined as an average systolic blood pressure of ≥140 mmHg, an average diastolic blood pressure of ≥90 mmHg, and/or current use of antihypertensive medications.

#### 2.2.2. Food and Nutrient Intake Assessment

Variables related to dietary intake were assessed based on the data collected using the 24-h recall method. In the KNHANES, trained dietitians conducted in-person interviews to estimate the dietary intake during the day prior by various measurement aids, such as two-dimensional models of the actual size of food, dishes, containers, measuring cups, spoons, 30 cm ruler, thickness sticks, tape measure, among others. In this study, total daily energy intake, percentage of energy intake from macronutrients (carbohydrate, protein, fat), and intake of dietary fiber, calcium, iron, and vitamin C were evaluated.

#### 2.2.3. KHEI

The KHEI is an indicator developed by the KCDC as a means of assessing adherence to national dietary guidelines and comprehensive dietary life and quality among Koreans [29]. It has a total of 14 components, eight evaluating the adequacy of the recommended food and nutrient intake (having breakfast, multi-grains, fruits, fresh fruits, vegetables, vegetables excluding kimchi and pickles, milk and dairy products, meat, fish, eggs, legumes), three evaluating the intake of foods and nutrients for which restriction of consumption is recommended (percentage of energy intake from saturated fatty acids, sugars, sweets, beverages, sodium), and three evaluating the balance of energy intake (percentage of energy intake from carbohydrates, fats, adequate energy intake) (Table S1 Supplementary). The maximum possible score of the KHEI is 100 points. Some of the components weigh 5 points (fruits, vegetables, multi-grains, percentage of energy intake from carbohydrates and fats, adequate energy intake), and the rest weigh 10 points given their importance. The KHEI score for each component was outlined in the KNHANES raw data.

### 2.3. Statistical Analysis

All statistical analyses were performed using SAS software, and significance was set at *p* < 0.05. With the characteristics of the KNHANES data, a complex sample design was used, applying kstrata and psu variables, as well as sample weights to data analysis. For each age group, socioeconomic variables, nutrient intake, and KHEI scores were presented as frequencies and percentages, and the significance was tested using the chi-squared test. Continuous variables were shown as mean and standard error, and the *t*-test was performed through regression analysis.

The differences in average nutrient intake and KHEI scores by sex and age groups were compared after adjusting for age, education level, household income, exercise, marital, drinking, and smoking status. To analyze the association between KHEI scores by sex and age groups (tertile) and metabolic abnormalities (obesity, diabetes, hypertension), the odds ratios (ORs) and 95% confidence intervals (CIs) were calculated. Potential confounding factors, such as sex, age, education level, household income, energy intake, marital, drinking, smoking, and exercise status were adjusted for in the analysis.

## 3. Results

### 3.1. General Characteristics

A total of 1442 participants were included in this study, 475 men and 967 women. The analysis results of the socioeconomic characteristics, sex, and age groups are presented in Table 1. The average ages of men and women were 58.14 years and 67.20 years, respectively (data not shown). In both sexes, single-person households in the 40–64 age group were significantly more likely to have college degrees or higher (*p* < 0.0001 for both), high household incomes (*p* < 0.0001 for both), be married (*p* < 0.0001 for both), currently smoke (*p* < 0.0001 for both), and drink (*p* = 0.0077 and *p* < 0.0001, respectively) than those in the 65 years and older age group.

There was no significant difference in the distribution of obesity based on BMI between the age groups of men aged 40 years and older. However, women aged 40–64 years were significantly less likely to be overweight or obese compared to those aged 65 years and older (*p* = 0.0043). In addition, among women living in single-person households, the 40–64-year-old group was significantly more likely to exercise by walking than those in the 65 years and older group (*p* = 0.0106).

### 3.2. KHEI

The analysis results of the KHEI score differences by sex and age groups are presented in Table 2. Among men, there was no significant difference in the total KHEI score by age. However, men aged 40–64 years had a significantly higher KHEI score for the total vegetable intake compared to those aged 65 and older (*p* = 0.0494). Among women, the 40–64 age group had a total KHEI score of 65.92, which was significantly lower than the 70.66 in the age group of 65 years and older (*p* = 0.0152). Moreover, in women, the total points of component adequacy were 31.95 in the 40–64 age group, which was significantly lower than the 36.76 in the 65 years and older age group (*p* = 0.0011). The same results were obtained in the component for total fresh fruit (*p* = 0.0185), vegetable (*p* = 0.0112), vegetable excluding kimchi and pickles (*p* = 0.0005), meat, fish, eggs, and legumes (*p* = 0.0011) intake. Among both men and women, an adequate intake of multi-grains, milk, and dairy products, a moderate intake of sodium, and a balanced carbohydrate energy intake scored the lowest.

### 3.3. Nutrient Intake

The analysis results of the differences in major nutrient intake by sex and age groups are presented in Table 3. Considering the characteristic differences by age groups, neither men nor women showed significantly different energy intake. In addition, the proportions of carbohydrate, protein, and fat intake did not significantly differ. However, among men, the intake of dietary fiber in the 40–64 age group was significantly higher than that of the 65 years and older group (*p* = 0.0388). Among women, the vitamin C intake in the 40–64 age group was significantly lower than that of the 65 years and older group (*p* = 0.0226).

### 3.4. Association between KHEI and Metabolic Abnormalities

The analysis results of the relationship between the KHEI and metabolic abnormalities (obesity, diabetes, hypertension) are presented in Table 4. Since age is one of the most important factors for metabolic abnormalities, all the study subjects were divided into two age groups (40–64 years and 65 years and older) and confounding factors, including education level, household income, marital, drinking, smoking, and exercise status, were adjusted for. Among men aged 65 years and older, the OR of diabetes in the T1 group was 2.16 times higher than the T3 group (95% CI: 1.03–4.51), classified according to the KHEI. Among all the participants aged 65 years and older of both sexes, the OR of diabetes in the T1 group was 2.08 times higher than the T3 group (95% CI: 1.36–3.17).

## 4. Discussion

In this nationally representative cross-sectional study that was conducted in Korea, individuals living in single-person households over the age of 40 years were divided into two groups (40–64 years and 65 years and older). Then, their overall dietary habits and quality were assessed, and sex differences were observed in the analysis. Among men living in single-person households, those aged 65 and older had only one significantly lower score, which was the total of vegetable intake, a component in the adequacy domain. Meanwhile, among women living in single-person households, those aged 65 and older had a significantly higher total KHEI score as well as higher scores for adequacy components, such as vegetables and protein. In addition, among single-person households aged 65 years and older, the odds of diabetes significantly increased in the participants with low KHEI scores. Most of the previous studies on diet and metabolic diseases focused only on the elderly, both adults and the elderly, or the intake of nutrients and foods [4,8,13]. However, this study identified dietary factors that may be associated with adverse health outcomes in single-person households specifically by sex and age groups through examining the overall dietary life and quality of individuals over 40 years of age, classified into two groups. To the best of our knowledge, this is the first study in which meaningful results were yielded regarding the association between metabolic abnormalities and the KHEI, a model that has been developed and statistically validated.

Single-person households are associated with unique nutritional characteristics. Since they are responsible for managing their own dietary lifestyles, such as preparing and consuming food, it depends on their perception and attitude towards dietary life, interest in health, and health management. Most recent studies have examined the nutritional and health status of the elderly who are living alone. Unlike middle-aged adults who are socially active, the elderly experience changes in socio-economic status, such as a reduction in household income, decline in social networks, and deterioration of body functions, which eventually lead to lower diet quality and increased risk for many chronic diseases. Therefore, the health and nutritional status of the elderly living alone has been studied thoroughly. However, according to a recent survey in Korea, those in their third decade accounted for 18.6% of the total single-person households, followed by those in their fifth (16.7%) and fourth (16.4%) decades, indicating a relatively high percentage of single-person households in these groups [30]. Adults in their fourth and fifth decade account for a high proportion of those participating in social activities and are exposed to excessive stress, reduced physical activity, and an unhealthy food environment. In addition, the prevalence of chronic diseases increases among adults in this age group.

Therefore, this study analyzed the dietary lifestyle of adults over 40 years and the elderly living alone, finding generally higher KHEI scores among elderly women compared to men. Many previous reports have provided evidence on this matter. In a study of Japanese elderly individuals aged 65–90 years, women had significantly higher dietary diversity scores [24]. Another study of adults aged 53 years and older in the United States reported that women scored significantly higher on fruit and vegetable intake [31]. A person’s nutritional status is closely related to the type and amount of food consumed, and handling and cooking skills are necessary to prepare healthy meals. According to Lavelle et al., participants with high diet quality had more confidence in their food and cooking skills, consuming significantly less takeaway food [32]. Additionally, several earlier studies described that women spend more time cooking and strive to develop skills [33,34], explaining why they had higher KHEI scores in our study.

This study also found that elderly women had significantly higher KHEI scores than younger ones. This result is consistent with previous studies that showed a high diet quality among older adults [35,36,37]. Our results also showed that the components with relatively low scores were mixed grains, fruits, fresh fruits, milk, and dairy product intake, as well as the percentage of energy from carbohydrates (data not shown). This goes along with the previous KHEI analysis of 15,954 adults using the KNHANES data of 2013–2015 [38]. This could be related to the fact that, among the participants living alone, consuming a variety of fresh foods can be rather difficult.

Food consumption is the daily process of supplying all the necessary nutrients for the body to function properly. Balanced meals ensure adequate micronutrient intake without an excess or deficiency of specific nutrients, and they have been closely associated with the onset of chronic conditions, such as cardiovascular disease and obesity [39,40]. Moreover, a balanced intake of nutrients and various foods is recommended in many countries. Hence, studies on the correlation between meal quality and health outcomes, such as obesity, diabetes, and hypertension, have been reported. However, since each country and ethnic group has its own meal patterns and most frequently consumed foods and dietary behaviors, it is very important to use a quality index based on the actual dietary life of the participants. In our study, we used the KHEI, an indicator developed based on dietary lifestyles in Korea, to assess the adherence to national guidelines and to evaluate the overall dietary life and quality among Koreans by scoring index components. As an indicator, the validity of the KHEI was verified [29], and higher scores correspond to a healthier diet. Many prior reports have exhibited a strong association between the KHEI and chronic diseases. A study on adults aged 20–64 years using the KNHANES 2013–2017 data demonstrated that the KHEI scores were inversely related to the risk of metabolic syndrome by 0.98-fold after adjusting for covariates and that a healthy diet high in calcium and vitamin C was specifically linked to a reduced risk of metabolic syndrome in both men and women [41]. In another study of 4107 adults aged 40–79 years, it was reported that a 10-point increase in the KHEI increased the rate of blood pressure control by 1.10 times (95% CI: 1.01–1.20) [42]. In a study by Park et al. of adults aged 20–64 years [43], the ratio of those with a healthy estimated cardiovascular age was 1.27 times higher in the group with a high KHEI score (95% CI: 1.09–1.49). Moreover, according to a meta-analysis of the relationship between the Healthy Eating Index (HEI), the Alternate Healthy Eating Index (AHEI), and the Dietary Approaches to Stop Hypertension (DASH), and the health outcomes among non-Koreans, diets that score highly on the HEI and AHEI significantly reduced the risk of type 2 diabetes mellitus [44]. Meanwhile, there has been no study reported on the association between diet quality index and diabetes in single-person households, thus making it difficult to compare results with the current study. In our study, the relationship between the KHEI and the health outcomes of chronic diseases in the middle-aged and the elderly in single-person households was analyzed, resulting in a significant negative association between the KHEI and diabetes only among those aged 65 years and older after adjusting for all the covariates.

This study has several limitations. Firstly, because of the sectional design of the KNHANES, causal inferences cannot be made. However, the KNHANES uses a rolling sampling design for participant selection, thus closely representing the Korean population. Secondly, a limited number of variables were used for living alone. For example, the elderly who lived alone may have had poor social networks, which are strongly related to health outcomes [45]. Nevertheless, to the best of our knowledge, this is the first study to systematically examine the relationship between the dietary lifestyle and health outcomes among middle-aged and elderly individuals in single-person households using an indicator that has been previously validated. In addition, single-person households were classified by age group to investigate the characteristics of dietary life and health outcomes, which can be used to identify significant dietary factors for specific chronic diseases.

## 5. Conclusions

This study found that the risk of diabetes significantly increases with decreasing diet quality. Given the risk of chronic diseases to which the elderly are unavoidably exposed, it is necessary to improve the overall diet quality among those in single-person households with a focus on the national dietary guidelines. However, it is challenging for the elderly living alone to prepare their own meals. Since the coronavirus disease 2019 outbreak, social activities and assistance, such as food distribution at senior centers, have been reduced, which is detrimental to improving their diet. Therefore, it is necessary to establish various policies to enhance the diet quality of the elderly living alone using our study as baseline data. The intake of home meal replacement (HMR) with enhanced nutrients and functional ingredients among the policies for improving the health of the elderly can also be used as an important means. Further research is needed to identify the various changing social characteristics of single-person households. For this purpose, the researchers will study the effects of varying HMR intake of the elderly living alone on disease improvement and mitigation.

## Figures and Tables

**Table 1:** General characteristics of study population in single-person households according to age group.

		Men (*n* = 475)			Women (*n* = 967)		
		40~64 Years	≥65 Years	*p*-Value	40~64 Years	≥65 Years	*p*-Value
Age(years)		51.13 ± 0.66	73.26 ± 0.40	<0.0001	54.74 ± 0.56	74.46 ± 0.20	<0.0001
Education level	≤Middle school	85 (23.44)	139 (64.83)	<0.0001	129 (40.37)	594 (87.40)	<0.0001
	High school	95 (40.27)	43 (20.86)		105 (34.98)	49 (8.72)	
	≥College	82 (36.29)	31 (14.31)		71 (24.65)	19 (3.88)	
Household income	Low	66 (21.35)	141 (64.56)	<0.0001	97 (32.37)	528 (78.68)	<0.0001
	Middle-low	69 (21.41)	44 (21.07)		109 (35.19)	104 (15.94)	
	Middle-high	46 (22.25)	17 (8.89)		56 (17.54)	20 (3.41)	
	High	81 (34.98)	11 (5.47)		43 (14.90)	10 (1.98)	
Marital status	Married	104 (44.58)	9 (4.35)	<0.0001	53 (19.65)	11 (1.87)	<0.0001
	Single	158 (55.42)	204 (95.65)		252 (80.35)	651 (98.13)	
Obesity degree	Underweight	10 (2.99)	10 (5.60)	0.2897	12 (4.30)	18 (2.8)	0.0043
	Normal weight	90 (34.84)	82 (39.77)		120 (39.05)	195 (29.42)	
	Overweight	80 (32.02)	51 (25.06)		79 (24.99)	150 (23.67)	
	Obesity	82 (30.15)	70 (29.58)		94 (31.66)	299 (44.11)	
Current smoking	No	108 (40.98)	155 (72.79)	<0.0001	272 (88.76)	641 (97.51)	<0.0001
	Yes	154 (59.02)	57 (27.21)		31 (11.24)	16 (2.49)	
Current drinking	No	82 (28.58)	86 (41.00)	0.0077	175 (56.05)	535 (80.39)	<0.0001
	Yes	180 (71.42)	127 (59.00)		129 (43.95)	122 (19.61)	
Physical(walking)	No	168 (63.49)	135 (59.02)	0.3611	176 (56.93)	454 (66.50)	0.0106
	Yes	94 (36.51)	75 (40.98)		129 (43.07)	202 (33.50)	

Data represent mean ± standard error or number of case (%).

**Table 2:** KHEI of study population in single-person households according to age group.

			Men (*n* = 475) ^1^			Women (*n* = 967) ^2^		
		Score	40~64 Years	≥65 Years	*p*-Value	40~64 Years	≥65 Years	*p*-Value
Adequacy	Have breakfast	0–10	8.33 ± 0.41	6.98 ± 0.46	0.0689	8.29 ± 0.43	8.34 ± 0.30	0.9009
	Mixed grains intake	0–5	1.19 ± 0.23	1.99 ± 0.26	0.0523	2.11 ± 0.29	1.95 ± 0.21	0.6141
	Total fruits intake	0–5	2.22 ± 0.25	2.00 ± 0.26	0.5876	2.56 ± 0.30	3.33 ± 0.21	0.0185
	Fresh fruits intake	0–5	2.35 ± 0.27	2.10 ± 0.28	0.5663	2.85 ± 0.29	3.18 ± 0.22	0.2734
	Total vegetables intake	0–5	4.27 ± 0.14	3.77 ± 0.16	0.0494	2.84 ± 0.20	3.38 ± 0.15	0.0112
	Vegetables intake excluding Kimchi and pickled vegetables intake	0–5	3.56 ± 0.18	3.27 ± 0.18	0.3350	2.77 ± 0.20	3.55 ± 0.16	0.0005
	Meat, fish, eggs and beans intake	0–10	6.75 ± 0.40	7.75 ± 0.42	0.1641	6.36 ± 0.44	7.97 ± 0.31	0.0011
	Milk and milk products intake	0–10	3.11 ± 0.45	3.18 ± 0.52	0.9206	4.16 ± 0.61	5.05 ± 0.49	0.1505
	Total scores of the adequacy	0–55	31.78 ± 1.18	31.05 ± 1.32	0.7151	31.95 ± 1.42	36.76 ± 1.00	0.0011
Moderation	% of energy from saturated fatty acid	0–10	7.80 ± 0.42	7.94 ± 0.42	0.8355	8.00 ± 0.45	7.55 ± 0.39	0.3438
	Sodium intake	0–10	5.92 ± 0.34	6.07 ± 0.37	0.7972	8.30 ± 0.33	8.47 ± 0.29	0.6643
	% of energy from sweets and beverages	0–10	9.59 ± 0.27	8.91 ± 0.32	0.1771	9.07 ± 0.26	9.07 ± 0.23	0.9986
	Total scores of the moderation	0–30	23.31 ± 0.61	22.92 ± 0.66	0.7095	25.37 ± 0.58	25.09 ± 0.54	0.7035
Balance of energy intake	% of energy from carbohydrate	0–5	2.18 ± 0.26	2.70 ± 0.27	0.2353	2.43 ± 0.27	2.63 ± 0.21	0.5120
	% of energy from fat	0–5	2.96 ± 0.26	3.46 ± 0.28	0.2747	3.36 ± 0.29	3.39 ± 0.21	0.9297
	Energy intake	0–5	3.29 ± 0.25	3.40 ± 0.27	0.7992	2.81 ± 0.34	2.80 ± 0.25	0.9750
	Total scores of the energy intake	0–15	8.44 ± 0.56	9.57 ± 0.50	0.1981	8.60 ± 0.62	8.82 ± 0.51	0.7542
Total scores of KHEI		0–100	63.53 ± 1.34	63.53 ± 1.47	0.9998	65.92 ± 1.81	70.66 ± 1.29	0.0152

Data represent mean ± standard error. ^1^ Adjusted by age, education level, household income, marriage, marital status, smoking status, and drinking status. ^2^ Adjusted by age, education level, household income, marriage, marital status, smoking status, drinking status, and physical activity (walking).

**Table 3:** Major intake calculated from 24-h recall method of study population in single-person households according to age group.

	Men (*n* = 475)			Women (*n* = 967)		
	40~64 Years	≥65 Years	*p*-Value	40~64 Years	≥65 Years	*p*-Value
Energy (kcal/day)	2202.39 ± 85.66	2086.52 ± 84.43	0.3974	1542.4 ± 75.51	1592.7 ± 63.74	0.5627
Fat (% Energy)	17.97 ± 1.14	19.15 ± 1.09	0.5117	19.17 ± 1.24	19.43 ± 0.98	0.8391
Carbohydrate (% Energy)	67.06 ± 1.41	65.24 ± 1.40	0.4207	66.20 ± 1.66	66.25 ± 1.30	0.9777
Protein (% Energy)	14.97 ± 0.55	15.62 ± 0.63	0.5172	14.63 ± 0.66	14.32 ± 0.45	0.5712
Fiber (g/day)	30.75 ± 1.75	24.88 ± 1.59	0.0388	25.23 ± 1.99	25.62 ± 1.89	0.8542
Calcium (mg/day)	607.85 ± 36.57	535.77 ± 47.66	0.3168	447.45 ± 44.13	510.79 ± 38.69	0.1776
Iron (mg/day)	13.74 ± 0.67	13.17 ± 0.93	0.6515	10.98 ± 0.68	10.75 ± 0.78	0.8189
Vitamin C (mg/day)	78.51 ± 13.20	50.71 ± 8.68	0.0031	51.60 ± 6.90	71.02 ± 6.91	0.0226

Data represent mean ± standard error.

**Table 4:** Adjusted odds ratios with 95% confidence intervals for obesity, diabetes, and hypertension by KHEI score among single-person households in Korea.

			Tertile of KHEI			
			T1	T2	T3	*p*-Value
			AOR (95% CI)	AOR (95% CI)		
Total	40–64 years	Obesity	0.87 (0.53–1.45)	0.90 (0.58–1.38)	1.00 (ref.)	0.8412
		Diabetes	0.84 (0.50–1.42)	0.82 (0.52–1.29)	1.00 (ref.)	0.6561
		Hypertension	0.82 (0.50–1.37)	1.15 (0.78–1.71)	1.00 (ref.)	0.3176
	≥65 years	Obesity	1.08 (0.77–1.51)	1.00 (0.72–1.38)	1.00 (ref.)	0.8896
		Diabetes	2.08 (1.36–3.17)	1.02 (0.71–1.47)	1.00 (ref.)	0.0004
		Hypertension	1.19 (0.78–1.81)	1.06 (0.72–1.58)	1.00 (ref.)	0.6975
Men	40–64 years	Obesity	0.84 (0.44–1.59)	0.88 (0.49–1.57)	1.00 (ref.)	0.8350
		Diabetes	0.88 (0.44–1.75)	1.13 (0.60–2.12)	1.00 (ref.)	0.7836
		Hypertension	0.63 (0.31–1.30)	1.01 (0.55–1.84)	1.00 (ref.)	0.3407
	≥65 years	Obesity	1.00 (0.48–2.08)	1.71 (0.82–3.57)	1.00 (ref.)	0.2724
		Diabetes	2.16 (1.03–4.51)	1.67 (0.78–3.59)	1.00 (ref.)	0.1218
		Hypertension	1.01 (0.42–2.43)	0.76 (0.32–1.80)	1.00 (ref.)	0.7320
Women	40–64 years	Obesity	0.64 (0.35–1.14)	0.77 (0.44–1.35)	1.00 (ref.)	0.3094
		Diabetes	1.12 (0.58–2.18)	1.63 (0.88–3.05)	1.00 (ref.)	0.2632
		Hypertension	0.98 (0.52–1.84)	0.67 (0.40–1.10)	1.00 (ref.)	0.1923
	≥65 years	Obesity	1.02 (0.68–1.51)	0.80 (0.53–1.20)	1.00 (ref.)	0.4251
		Diabetes	1.61 (0.98–2.65)	0.98 (0.64–1.49)	1.00 (ref.)	0.0579
		Hypertension	1.16 (0.73–1.86)	1.05 (0.65–1.69)	1.00 (ref.)	0.8007

Adjusted for by age, education level, household income, marriage, marital status, smoking status, drinking status, physical activity(walking), and energy intake; AOR, adjusted odds ratio; CI, confidence interval.

## Data Availability

The KNHANES data used in the manuscript can be found at the following link: https://knhanes.kdca.go.kr/knhanes/main.do (accessed on 21 July 2021).

## Supplementary Materials

The following are available online at https://www.mdpi.com/article/10.3390/nu13113937/s1: Table S1: Korean Healthy Eating Index components and standards for scoring.
