# Supplementary material for: Association of the Healthy Eating Index with Metabolic Abnormalities among Middle-Aged Adults Living in Single-Person Households in Korea
Source: Nutrients. 2021 Nov 4;13(11):3937. doi: 10.3390/nu13113937 (PMC8624069; doi:10.3390/nu13113937)
Supplement: Supplementary file 1 [file nutrients-13-03937-s001.zip › nutrients-1399334-supplementary.pdf]

Table S1 Supplementary. Korean Healthy Eating Index components and standards for scoring

| Classification (No. of components) | Components | Score range | Standard for maximum score | Standard for minimum score |
|---|---|---|---|---|
| Adequacy (8) | Have breakfast* | 0-10 | 5-7 times/w | 0 times/w |
| | Mixed grains intake*,† | 0-5 | ≥ 0.3 serving/d | 0 serving/d |
| | Total fruits intake† | 0-5 | • Men aged 19-64 years: ≥ 3 serving/d<br>• Men aged 65 years and overs: ≥ 2 serving/d<br>• Women aged 19-64 years: ≥ 2 serving/d<br>• Women aged 65 years and overs: ≥ 1 serving/d | 0 serving/d |
| | Fresh fruits intake*,† | 0-5 | • Men aged 19-64 years: ≥ 1.5 serving/d<br>• Women aged 19-64 years: ≥ 1 serving/d<br>• Men aged 65 years and overs: ≥ 1 serving/d<br>• Women aged 65 years and over: ≥ 0.5 serving/d | 0 serving/d |
| | Total vegetables intake† | 0-5 | • Men and women aged 19-64 years: ≥ 8 serving/d<br>• Men aged 65 years and overs: ≥ 8 serving/d<br>• Women aged 65 years and overs: ≥ 6 serving/d | 0 serving/d |
| | Vegetables intake excluding Kimchi and pickled vegetables intake*,† | 0-5 | • Men and women aged 19-64 years: ≥ 5 serving/d<br>• Men aged 65 years and overs: ≥ 5 serving/d<br>• Women aged 65 years and overs: ≥ 3 serving/d | 0 serving/d |
| | Meat, fish, eggs and beans intake† | 0-10 | • Men aged 19-64 years: ≥ 5 serving/d<br>• Women aged 19-64 years: ≥ 4 serving/d<br>• Men aged 65 years and overs: ≥ 4 serving/d<br>• Women aged 65 years and overs: ≥ 2.5 serving/d | 0 serving/d |
| | Milk and milk products intake† | 0-10 | ≥ 1 serving/d | 0 serving/d |
| Moderation (3) | Percentage of energy from saturated fatty acid†,‡ | 0-10 | ≤ 7% of total energy intake | > 10% of total energy intake |
| | Sodium intake†,§ | 0-10 | ≤ 2,000 mg/d | > 6,500 mg/d |
| | Percentage of energy from sweets and beverages† | 0-10 | ≤ 10% of total energy intake | > 20% of total energy intake |
| Balance of energy intake (3) | Percentage of energy from carbohydrate†,§ | 0-5 | 55-65% of total energy intake | < 50% or > 75% of total energy intake |
| | Percentage of energy intake from fat†,‡,§ | 0-5 | 15-30% of total energy intake | < 10% or > 35% of total energy intake |
| | Energy intake†,§ | 0-5 | 75-125% of the estimated energy intake requirement (EER) | < 60% or > 140% of EER |

*Dietary guidelines for Korean adults

†Dietary Reference Intake for Koreans 2015

‡Recommendation criteria of WHO/FAO

§15 or 85 percentile value in Korean adults aged 19 years and over

From Yook et al. J Nutr Health 2015, 48, 419-428. http://dx.doi.org/10.4163/jnh.2015.48.5.419
